# Inpatient Diagnosis of Delirium and Encephalopathy: Coding Trends in 2011-2018

**DOI:** 10.1093/geroni/igaa057.3329

**Published:** 2020-12-16

**Authors:** Jeffrey Franks, Jami Anderson, Richard Kennedy, Huifeng Yun

**Affiliations:** University of Alabama at Birmingham, Birmingham, Alabama, United States

## Abstract

Physicians have long debated the diagnosis of acute confusional states as delirium or encephalopathy, often based on specialty. Recently, CMS assigned a lower severity to the nonspecific behavioral diagnosis of delirium than for the pathophysiological diagnosis of encephalopathy, potentially exacerbating these disagreements. Therefore, we sought to evaluate trends in these two diagnoses among hospitalized adults. Using 2011-2018 IBM MarketScan datasets, we identified delirium/encephalopathy patients who were ≥ 18 years and enrolled with medical and pharmacy coverage for each calendar year. Delirium/encephalopathy were defined using validated ICD-9/10 codes among hospitalized patients. We identified the physician specialties associated with the hospitalization and comorbidities using ICD9/10 inpatient/outpatient diagnosis codes within one year prior to the diagnosis of delirium or encephalopathy. Log-binomial models were used to evaluate the trends adjusting for age, gender, insurance and comorbidities. We identified 10,418 delirium and 87,393 encephalopathy hospitalized patients in 2011-2018. Of these patients, the total number of patients with either diagnosis increased, but the proportion of patients with delirium for each year decreased from 20% in 2011 to 9% in 2018. During the 8 years, neurologists and internists increased their use of both diagnoses, whereas psychiatrists only increased for delirium. Patients with encephalopathy are more likely to be older, female, and have more comorbidities. These shifts in diagnosis complicate the study of delirium and encephalopathy, and can lead to erroneous conclusions about trends in the incidence and prevalence of these disorders unless properly understood.

